# OA17 Improvement in Sjögren’s disease-associated interstitial lung disease following immunosuppressive therapy: a case report

**DOI:** 10.1093/rap/rkag113.017

**Published:** 2026-11-03

**Authors:** Alexandra Godlee, Midhat Asif, Kevingulie Savino, Benjamin Fisher

**Affiliations:** University Hospitals Birmingham NHS Foundation Trust, Birmingham, United Kingdom; University Hospitals Birmingham NHS Foundation Trust, Birmingham, United Kingdom; University Hospitals Birmingham NHS Foundation Trust, Birmingham, United Kingdom; University Hospitals Birmingham NHS Foundation Trust, Birmingham, United Kingdom; University of Birmingham, Birmingham, United Kingdom

## Abstract

**Introduction:**

Primary Sjögren’s disease (SjD) is a chronic autoimmune disorder characterised by lymphocytic infiltration of the exocrine glands, resulting in sicca symptoms. Extra-glandular manifestations are common and contribute substantially to morbidity. Interstitial lung disease (ILD) is one of the most serious systemic complications, affecting 10 to 15 percent of patients, with nonspecific interstitial pneumonia (NSIP) representing the most frequent radiological pattern. Early recognition and timely immunosuppressive treatment have been shown to preserve lung function and improve outcomes. We present a case of SjD-associated ILD demonstrating sustained improvement in respiratory function following immunosuppressive therapy, including rituximab after azathioprine intolerance.

**Case description:**

Our patient was diagnosed with Sjögren’s disease (SjD) aged 28 after she developed sicca symptoms and dysphonia. ANA was positive at 1:1600 with a speckled pattern alongside positive anti-Ro, anti-La and rheumatoid factor. Ultrasound of the salivary glands showed diffuse autoimmune foci in keeping with SjD.

Several years after diagnosis she developed inflammatory arthritis which was treated with methotrexate and intermittent oral corticosteroids. She subsequently noticed worsening shortness of breath and was found to have a restrictive pattern on lung function testing and ground glass changes on computed tomography (CT) scan of the chest in keeping with nonspecific interstitial pneumonia (NSIP). She was diagnosed with Sjögren’s related interstitial lung disease. Immunosuppression was commenced with azathioprine and prednisolone with some improvement in lung function. During her second pregnancy azathioprine was withdrawn due to neutropaenia and her respiratory function declined. Rituximab was initiated with a rebound recovery in her respiratory function.

Following rituximab therapy the patient had an acute infectious illness requiring hospital admission. During this period she suffered sensorineural hearing loss in one ear and partial hearing loss in the other ear. Rituximab infusions were stopped with the aim of transitioning to mycophenolate.

In the seven years since diagnosis of ILD forced expiratory volume in 1 second (FEV1) has improved from 76% at diagnosis to 86%. Transfer factor of the lung for carbon monoxide (TLCO) has improved from 60% to 80%. The significant ground glass changes on CT scan have almost entirely resolved. Overall, her respiratory function has improved significantly with immunosuppression.

**Discussion:**

This case highlights the importance of recognising interstitial lung disease (ILD) as a significant extra-glandular manifestation of primary Sjögren’s disease (SjD). Although sicca symptoms are the hallmark of SjD, pulmonary involvement occurs in up to 20% of patients and is associated with increased morbidity and mortality. Nonspecific interstitial pneumonia (NSIP) is the most frequently reported radiological pattern and may present insidiously with progressive dyspnoea and declining pulmonary function. As symptoms can be subtle, a low threshold for investigating respiratory complaints with pulmonary function testing and high-resolution CT imaging is important to allow earlier diagnosis and treatment.

The management of SjD-associated ILD remains challenging because evidence is largely derived from observational studies and expert consensus rather than randomised controlled trials. Conventional immunosuppressive therapies, including corticosteroids, azathioprine and mycophenolate mofetil, are commonly used, although treatment responses are variable. In this case, initial improvement was achieved with azathioprine and corticosteroids; however, treatment was complicated by neutropenia during pregnancy, necessitating withdrawal of azathioprine and resulting in clinical deterioration. Subsequent treatment with rituximab was associated with improvement in the patient’s symptoms as well as measured by lung function and radiologically.

Although evidence for rituximab in Sjögren’s-associated ILD remains limited, this case supports its use in patients with progressive disease or intolerance to conventional immunosuppressive therapy. It also highlights the value of close collaboration between rheumatology and respiratory teams, with regular assessment of symptoms and pulmonary function to guide treatment decisions. Further prospective studies are needed to better define the role of biologic therapy in this patient group.

**Key learning points:**

1. Interstitial lung disease is an important extra-glandular manifestation of Sjögren’s disease and should be considered in patients with new or progressive respiratory symptoms.

2. Early diagnosis and appropriate immunosuppressive treatment can stabilise or improve pulmonary function, emphasising the importance of regular respiratory assessment and multidisciplinary management.

3. Rituximab may be an effective treatment option for Sjögren’s-associated ILD in patients with progressive disease or intolerance to conventional immunosuppressive therapy.

